# ChREBP-Mediated Regulation of Lipid Metabolism: Involvement of the Gut Microbiota, Liver, and Adipose Tissue

**DOI:** 10.3389/fendo.2020.587189

**Published:** 2020-12-03

**Authors:** Katsumi Iizuka, Ken Takao, Daisuke Yabe

**Affiliations:** ^1^ Department of Diabetes and Endocrinology, Gifu University Graduate School of Medicine, Gifu, Japan; ^2^ Center for Nutritional Support and Infection Control, Gifu University Hospital, Gifu, Japan; ^3^ Yutaka Seino Distinguished Center for Diabetes Research, Kansai Electric Power Medical Research Institute, Kobe, Japan; ^4^ Division of Molecular and Metabolic Medicine, Kobe University Graduate School of Medicine, Kobe, Japan

**Keywords:** fatty acid synthesis, lipoprotein metabolism, β-oxidation, ketogenesis, carbohydrate response element-binding protein (Chrebp), gut microbiota

## Abstract

Carbohydrate response element-binding protein (ChREBP) plays an important role in the development of type 2 diabetes, dyslipidemia, and non-alcoholic fatty liver disease, as well as tumorigenesis. ChREBP is highly expressed in lipogenic organs, such as liver, intestine, and adipose tissue, in which it regulates the production of acetyl CoA from glucose by inducing *Pklr* and *Acyl* expression. It has recently been demonstrated that ChREBP plays a role in the conversion of gut microbiota-derived acetate to acetyl CoA by activating its target gene, *Acss2*, in the liver. ChREBP regulates fatty acid synthesis, elongation, and desaturation by inducing *Acc1* and *Fasn*, elongation of long-chain fatty acids family member 6 (encoded by *Elovl6*), and *Scd1* expression, respectively. ChREBP also regulates the formation of very low-density lipoprotein by inducing the expression of *Mtp*. Furthermore, it plays a crucial role in peripheral lipid metabolism by inducin*g Fgf21* expression, as well as that of *Angptl3* and *Angptl8*, which are known to reduce peripheral lipoprotein lipase activity. In addition, ChREBP is involved in the production of palmitic-acid-5-hydroxystearic-acid, which increases insulin sensitivity in adipose tissue. Curiously, ChREBP is indirectly involved in fatty acid β-oxidation and subsequent ketogenesis. Thus, ChREBP regulates whole-body lipid metabolism by controlling the transcription of lipogenic enzymes and liver-derived cytokines.

## Introduction

**Figure 1:**
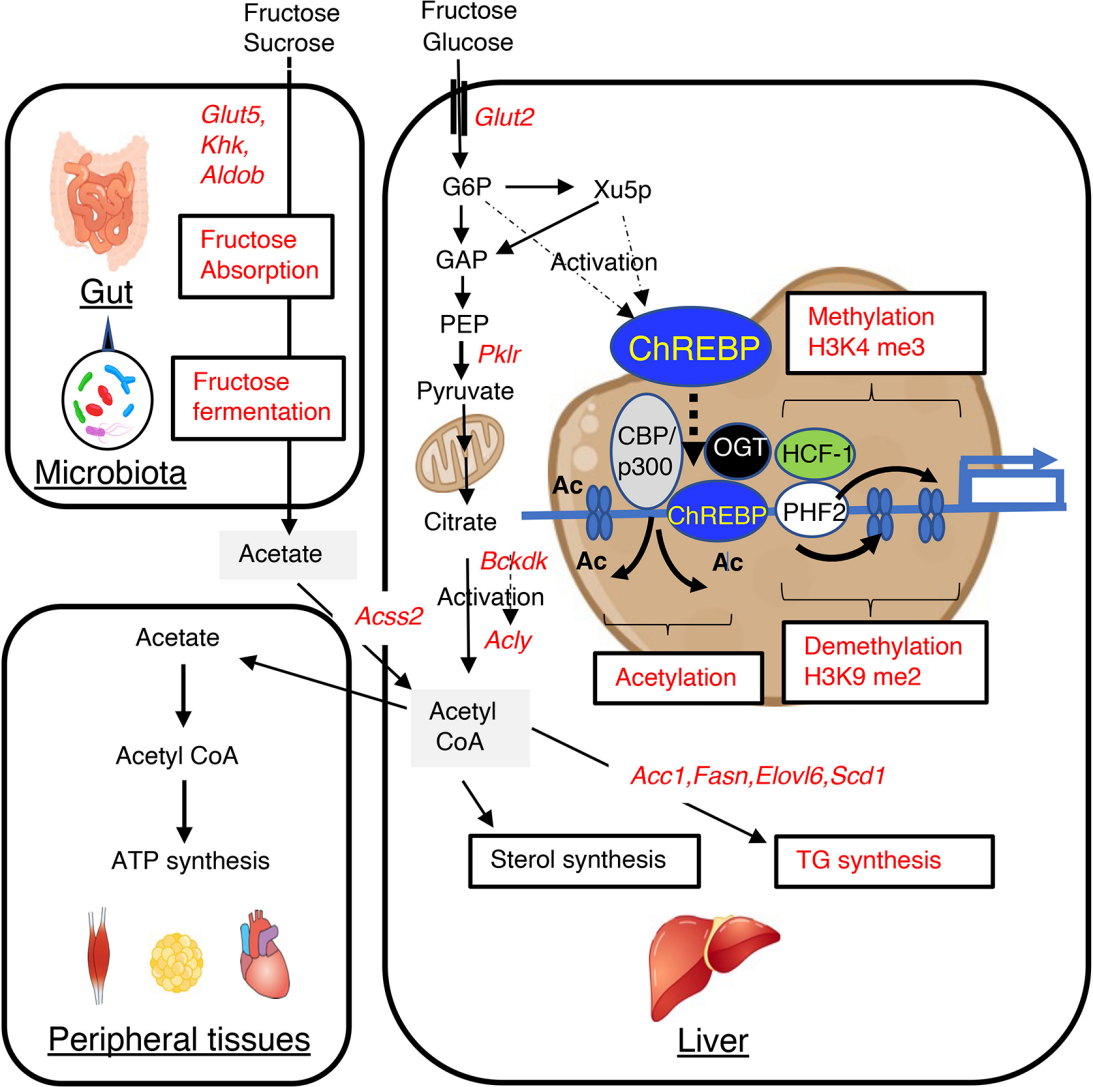
High-fructose/high-sucrose diet-feeding causes an increase in *de novo* lipogenesis through the regulation of lipogenic gene expression and gut microbiota-derived acetate utilization. Fructose is normally absorbed and converted to glucose and lactate. Glucose, lactate, and a small amount of fructose enter the portal vein. In the liver, glucose and fructose activate ChREBP transcriptional activity through increases in the concentrations of glucose and fructose-derived metabolites, such as xylulose-5-phosphate and glucose-6-phosphate, and this results in greater expression of lipogenic genes, such as *Acc1*, *Fasn*, *Elovl6*, and *Scd1*. This higher expression causes the metabolism of glucose and fructose to generate acetyl CoA and fatty acyl CoA in the liver. Fructose that is not absorbed in the small intestine is absorbed in the colon and enters the portal vein. In the liver, acetate is converted to acetyl CoA by acyl-coenzyme A synthetase short-chain family member 2 (encoded by *Acss2*), and this is used for fatty acyl CoA synthesis, sterol synthesis, and histone acetylation. ChREBP transcription activity is also regulated by acetyl CoA and uridine diphosphate-N-acetylglucosamine, through acetylation and O-GlcNAcylation, respectively. These substances are involved in epigenic regulation, such as histone acetylation and histone methylation. GLUT5, glucose transporter 5; KHK, ketohexokinase; ALDOB, aldolase B; GLUT2, glucose transporter 2; PKLR, liver-type pyruvate kinase; ACSS2, acyl-coenzyme A synthetase short-chain family member 2; G6P, glucose 6-phosphate; Xu5P, xylulose 5-phosphate; ACLY, ATP citrate lyase; ACC1, acetyl CoA carboxylase; FASN, fatty acid synthase; ELOVL6, fatty acid elongase 6; SCD1, stearyl CoA desaturase; BCKDK, branched-chain ketoacid dehydrogenase kinase; CBP, CREB binding protein; OGT, O-linked N-acetylglucosamine (GlcNAc) transferase; HCF-1, host cell factor-1; PHF2, plant homeodomain finger 2; H3K4me3, trimethylated H3K4; H3K9me2, dimethylated H3K9.

Excess carbohydrate intake causes hepatic triglyceride accumulation through the activation of carbohydrate response element binding protein (ChREBP) and *de novo* lipogenesis. Dietary carbohydrate is metabolized to acetyl CoA, which is a key intermediate in lipid metabolism (1). Acetyl CoA is produced by the oxidation of pyruvate, the end product of glycolysis, through the action of pyruvate dehydrogenase (PDH) and fatty acid β-oxidation, for use as a substrate in triglyceride and cholesterol synthesis, as well as ketogenesis and protein acetylation (1). In the fed state, the resulting high nucleocytosolic acetyl CoA is readily utilized for lipid synthesis and histone acetylation (1, 2). In contrast, in the fasted state or under more extreme conditions, acetyl CoA is preferentially directed to the mitochondria to permit greater synthesis of adenosine triphosphate (ATP) and ketone bodies (1, 2). Acetyl CoA is produced in the liver from glucose and fructose, which are rapidly converted to glyceraldehyde-3 phosphate (GAP) by glycolysis and fructolysis (**Figure 1**). GAP is then converted to pyruvate by several glycolytic enzymes, including liver-type pyruvate kinase (encoded by *Pklr*), which is activated by ChREBP and converts phosphoenolpyruvate to pyruvate (1). Pyruvate is then converted to acetyl CoA by the PDH complex in the mitochondria. When acetyl CoA enters the tricarboxylic acid cycle, there is an increase in the production of citrate, which is exported from the mitochondria and converted to acetyl CoA by ATP citrate lyase (encoded by *Acly*) (1, 2). Cytosolic acetyl CoA is converted into long-chain fatty acyl CoA by lipogenic enzymes such as acetyl CoA carboxylase 1 (encoded by *Acc1*) and fatty acid synthase (encoded by *Fasn*).

Carbohydrate response element-binding protein (ChREBP) plays a pivotal role in the pathogenesis of metabolic diseases and tumors (3–6). ChREBP was initially identified as a glucose-activated transcription factor that binds to the carbohydrate response element (ChoRE) in the promoter of *Pklr* (6). ChoREs are also found in the promoters of lipogenic genes, including *Acc1* and *Fasn* (7, 8), and ChREBP, together with its heterodimerization partner Max-like factor X, controls *de novo* lipogenesis in the liver by upregulating the expression of lipogenic genes (9–12). Studies that used DNA microarray, chromosome immunoprecipitation-sequencing (ChIP-seq) and *Chrebp* knockout mice have confirmed a critical role for ChREBP in hepatic glucose and lipid metabolism (13–16). Interestingly, ChREBP also modulates lipolysis in adipose tissue by directly regulating the expression of *Fgf21* and *Angptl* genes (14, 17–23). Furthermore, it regulates the hepatic and intestinal metabolism of fructose, which is tightly linked to lipogenesis and the pathogenesis of the metabolic syndrome (24–28). In addition, ChREBP plays a critical role in the conversion of gut microbiota-derived acetate, the production of which is increased by excess dietary fructose intake, to acetyl coenzyme A (CoA) by activating its target, *Acss2*, in the liver, which contributes to hepatic triglyceride accumulation (29). ChREBP also modulates hepatic β-oxidation and ketogenesis (30–32). This review focuses on recent advances in knowledge of the ChREBP-mediated regulation of lipid metabolism in the liver, adipose tissue, and gut.

## Carbohydrate Response Element-Binding Protein Regulates Hepatic *De Novo* Lipogenesis

### The Conventional Pathway for the Production of Acetyl Coenzyme A

As described in the Introduction, ACLY is an important mediator of the supply of acetyl CoA from glycolysis and fatty acid oxidation. Because the ACLY-mediated conversion of citrate to acetyl CoA is critical for the synthesis of triglycerides and sterols, efforts have been made to develop ACLY inhibitors for the treatment of hypertriglyceridemia and hypercholesterolemia (2, 3). *Acly* is expressed mainly in the liver and white adipose tissue and its expression is upregulated at the transcriptional level by glucose-activated ChREBP and the insulin-induced transcription factor sterol response element-binding protein-1c (SREBP-1c) (13, 14, 33, 34). The enzymatic activity of ACLY is post-translationally regulated by the phosphatidylinositol 3-kinase (PI3K)/Akt pathway and acetylation (35, 36). In addition, recent studies have demonstrated that ACLY is activated by branched-chain ketoacid dehydrogenase kinase (BDK) and mitochondrial protein phosphatase 1K (PPM1K), which are respectively positively and negatively regulated by ChREBP (37–39) (**Figure 1**). Furthermore, the branched-chain α-ketoacid dehydrogenase complex (BCKDH) is an enzyme that catalyzes the commitment step of branched-chain amino acid (BCAA) catabolism and is negatively and positively regulated by BDK and PPM1K, respectively. Thus, ChREBP regulates the use of BCAAs in triglyceride synthesis by regulating BDK/PPM1K (39). Therefore, ChREBP might contribute to the development of BCAA-induced insulin resistance (39).

### A Novel Pathway for the Production of Acetyl Coenzyme A

Recent studies have revealed that gut microbiota-derived acetate also represents an important source of acetyl CoA for hepatic lipogenesis (29). Dietary fructose is absorbed and metabolized to glucose in the intestine, whereas unabsorbed fructose is fermented by the gut microbiota to produce acetate (26, 27, 29, 40–42). Glucose induces the expression of both ChREBP*-*β and SREBP-1c, whereas fructose only induces the expression of SREBP-1c in the liver (28). Our group and others have recently reported that ChREBP regulates intestinal fructose absorption by inducing the expression of *Glut5*, *Khk*, and *Aldob* in the intestine and that *Chrebp* knockout mice consuming a sucrose-based diet show an irritable bowel syndrome-like phenotype, which develops because of fructose malabsorption and an increase in the numbers of acetate-producing bacteria in the intestine (26, 27). These findings suggest that the inhibition of intestinal ChREBP increases microbial acetate production by increasing the entry of unabsorbed fructose into the colon. Gut microbiota-derived acetate is absorbed and reaches the liver *via* the portal vein. Acetate is then converted to acetyl CoA by acyl-CoA synthetase short-chain family member, which is encoded by *Acss2*, and used as a substrate for lipogenesis (43, 44). *Acss2* is highly expressed in the kidney and liver and is present in both the cytosol and nucleus (45, 46). Importantly, depletion of the gut microbiota using antibiotics suppresses acetate production in the colon, and subsequent acetyl CoA synthesis and *de novo* lipogenesis in the liver; and the knock-down of *Acss2* mRNA using siRNA also reduces the hepatic production of acetyl CoA (29). In addition, it has been shown that SREBP-1c, a critical regulator of hepatic lipogenesis that is transcriptionally activated by insulin, induces *Acss2* mRNA expression (47–49). ChREBP also induces *Acss2* expression (**Figure 1**) (14, 29, 33). Thus, inhibition of hepatic ChREBP is likely to prevent fructose-induced triglyceride accumulation by suppressing lipogenic gene expression and hepatic acetyl CoA production from gut microbial acetate, while the inhibition of intestinal ChREBP may increase acetate production in the intestine (27).

### Fatty Acid Synthesis, Elongation, and Desaturation in the Liver

**Figure 2:**
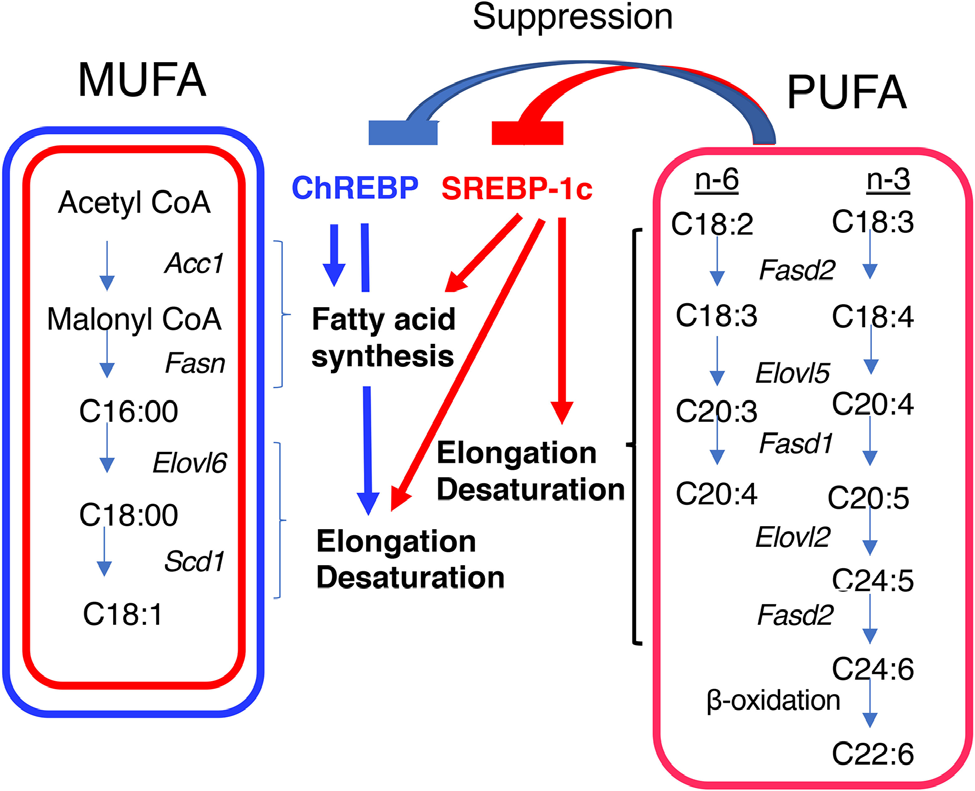
Differential regulation of monounsaturated fatty acid (MUFA) and polyunsaturated fatty acid (PUFA) synthesis by ChREBP and SREBP-1c. Both ChREBP and SREBP-1c regulate MUFA synthesis by regulating the expression of genes involved in fatty acid synthesis (e.g., *Acc1* and *Fasn*), elongation (e.g., *Elovl6*), and desaturation (e.g., *Scd1*); whereas SREBP-1c, but not ChREBP, regulates PUFA synthesis by regulating the expression of genes involved in elongation (e.g., *Elovl2*) and desaturation [e.g., fatty acid desaturase 1 (*Fasd1*) and fatty acid desaturase 2 (*Fasd2*)]. PUFAs suppress the transcriptional activities of ChREBP and SREBP-1c. ACC1, acetyl CoA carboxylase; FASN, fatty acid synthase; ELOVL6, fatty acid elongase 6; SCD1, stearyl CoA desaturase; ELOVL5, fatty acid elongase 5; FASD1, fatty acid desaturase 1; FASD2, fatty acid desaturase 2.

Acetyl CoA is converted to fatty acids through fatty acid synthesis, elongation, and desaturation; and the resulting fatty acids are then esterified with glycerol before being packaged into very-low-density lipoprotein (VLDL) particles to be delivered to the periphery (50). These processes are transcriptionally regulated by ChREBP and SREBP-1c (51) (Table 1). In fatty acid synthesis, both ChREBP and SREBP-1c induce *Acc*, which encodes an enzyme that is required for the conversion of acetyl CoA to malonyl CoA, and *Fasn*, which encodes an enzyme that is required for the production of palmitoyl CoA from malonyl CoA and acetyl CoA (9, 10, 13, 34, 51). In fatty acid elongation and desaturation, ChREBP induces the expression of the stearoyl CoA desaturase-1 gene (*Scd-1*) and *Elovl6*, both of which are required for the synthesis of monounsaturated fatty acids (MUFAs), while SREBP-1c induces *Elovl2*, fatty acid desaturase 1, and fatty acid desaturase 2, which are required for the synthesis of polyunsaturated fatty acids (PUFAs), as well as *Scd-1* and *Elovl6* (**Figure 2**) (52–56). Consistent with this, hepatic *Chrebp* overexpression reduces the concentrations of saturated fatty acids (SFAs), such as palmitic acid (C16:0) and stearic acid (C18:0); and increases that of the MUFA oleic acid (C18:1 n-9) (32, 57). Furthermore, hepatic SREBP-1c overexpression reduces the concentration of stearic acid (C18:0) and increases that of oleic acid (C18:1 n-9) (58). Importantly, PUFAs suppress the transcriptional activities of both ChREBP and SREBP-1c (59, 60). Moreover, PUFAs also reduce the cleavage of SREBP-1c at the post-transcriptional level (61). Thus, both ChREBP and SREBP-1c regulate *de novo* lipogenesis, elongation, and desaturation.

### Carbohydrate Response Element Binding Protein-Mediated Epigenetic Regulation of Lipogenic Gene Expression

The transcriptional activity of ChREBP is activated by glucose-derived metabolites such as glucose-6-phosphate, xylulose-5-phophate, and fructose-2,6-bisphosphate *via* regulation of its nucleocytoplasmic translocation (62–65). Moreover, ChREBP transcription activity is also regulated by acetyl CoA and uridine diphosphate-N-acetylglucosamine, through acetylation and O-GlcNAcylation, respectively (66–73). These substances are involved in epigenic regulation, such as histone acetylation and histone methylation (**Figure 1**).

During weaning, *Fasn* mRNA expression is increased by greater binding of ChREBP and SREBP-1c to *Fasn* promoter and greater histone H3 and H4 acetylation (70). Moreover, ChREBP induces *Fasn* mRNA expression and triglyceride synthesis by facilitating H3 and H4 acetylation, but these effects are prevented by the inhibition of histone acetylation using garcinol, a histone acetyltransferase (HAT) inhibitor (71). The role of the histone deacetylase activity of CREB-binding protein (CBP)/p300 in the mechanism of ChREBP-induced histone acetylation is well known (72). In human hepatocytes, the binding of farnesoid X receptor (FXR) to the ChREBP- Hepatocyte Nuclear Factor 4α (HNF4α) complex triggers the release of ChREBP from CBP/p300, leading to the recruitment of the histone deacetylase, silencing mediator of retinoic acid and thyroid hormone receptor (SMRT), to the *Lpk* promoter, where it acts as a co-repressor of ChREBP transcriptional activity (73). Interestingly, CBP/p300 HAT acetylates ChREBP, which promotes transactivation by this molecule (72). Therefore, CBP/p300 regulates ChREBP transcriptional activity through the acetylation of substrates such as histones and ChREBP (**Figure 1**).

Glucose activates the hexosamine pathway and thereby O-linked N-acetylglucosamine transferase (OGT)-mediated ChREBP O-GlcNacylation, which enhances ChREBP DNA-binding and protein stability by reducing ubiquitin-mediated degradation (66–68). Recently, host cell factor-1 (HCF-1) has been identified as a ChREBP-interacting protein (74). Glucose stimulates HCF-1 O-GlcNAcylation and cleavage, and interacts with ChREBP, thereby augmenting ChREBP O-GlcNAcylation and the recruitment of OGT to ChREBP (74). HCF-1 also augments the trimethylation of H3K4, which promotes the recruitment of the histone demethylase, PHD Finger Protein 2 (PHF2), resulting in greater transcriptional activity (74). PHF2 is also known to be a coactivator of ChREBP, and the ChREBP-PHF2 interaction causes the expression of *Scd1* and MUFA synthesis through H3K9me2 demethylation (75). H3K4 methylation and H3K9me2 contribute to transcriptional activation and repression, respectively. Thus, ChREBP and its co-factors, such as HCF-1, PHF2, and OGT, also regulate lipogenic gene expression through epigenetic modifications (**Figure 1**).

## The Regulation of Lipoprotein Metabolism by Carbohydrate Response Element Binding Protein

**Figure 3:**
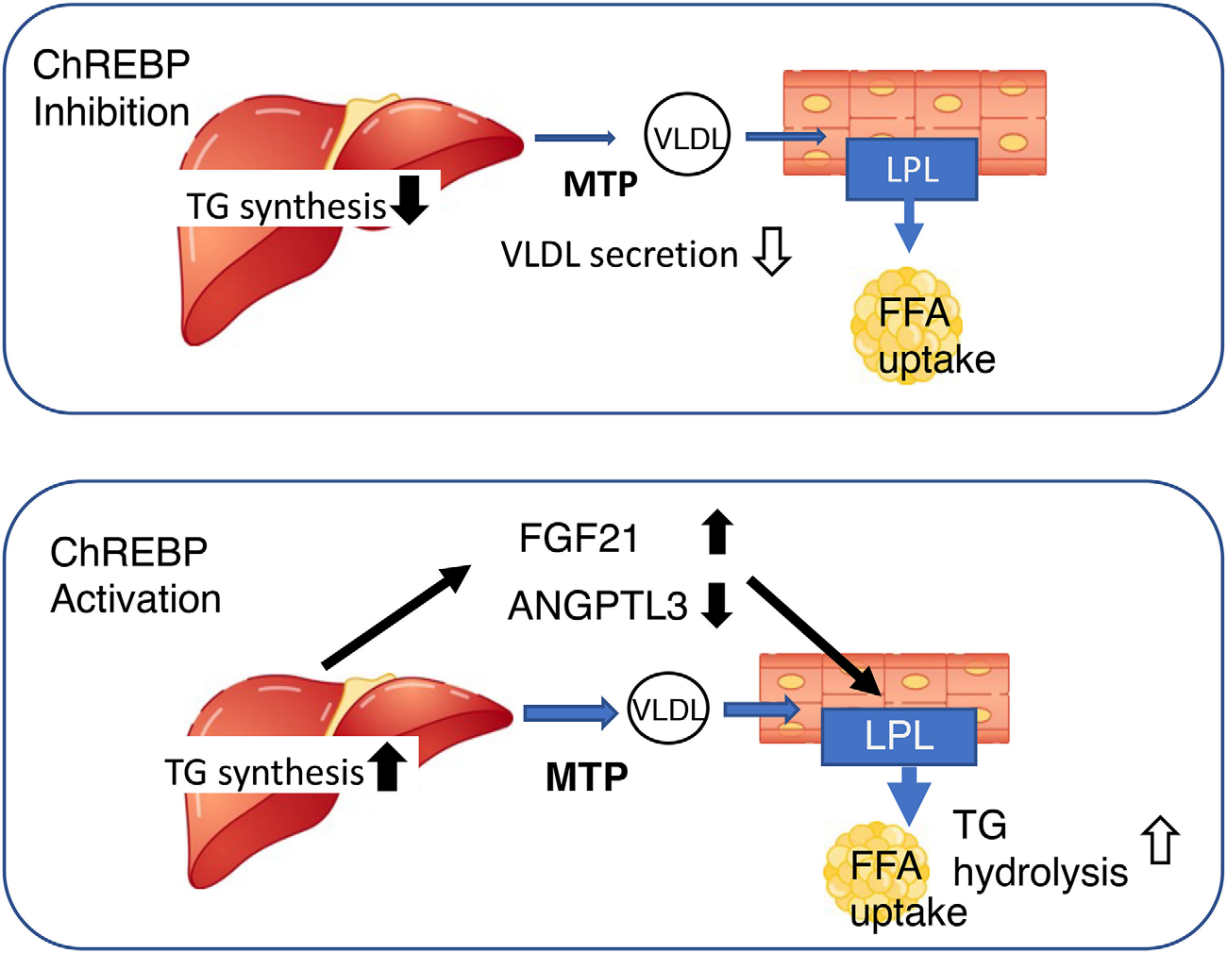
Both the activation and inhibition of ChREBP reduce blood triglyceride concentration, but through differing mechanisms. *Upper*, ChREBP inhibition reduces *de novo* lipogenesis and very-low density lipoprotein (VLDL) formation, resulting in lower circulating triglyceride concentration. *Lower*, ChREBP activation increases hepatic triglyceride synthesis, increases *Fgf21* expression, and reduces *Angptl3* expression. High FGF21 and low ANGPTL3 concentrations increase lipoprotein lipase activity in adipose tissue, thereby reducing the circulating triglyceride concentration. FGF21, fibroblast growth factor 21; MTTP, microsomal triglyceride transfer protein; LPL, lipoprotein lipase; TG, triglyceride; FFA, free fatty acid; ANGPTL3, angiopoietin-like 3.

It has been suggested that ChREBP may regulate lipoprotein metabolism, because the plasma concentrations of cholesterol, triglyceride, and very low density lipoprotein (VLDL)-triglyceride, as well as the number of VLDL particles, are lower in *Chrebp* knockout mice (13, 31, 58, 76). Our detailed analysis demonstrated that the mRNA and protein expression of hepatic microsomal triglyceride transfer protein (MTP) is lower in these mice (58, 76). MTP catalyzes the rate-limiting step in the production of apoB-containing VLDL, and therefore plays an important role in VLDL secretion (77). Furthermore, the consumption of a high fat/high sucrose diet is associated with lower VLDL secretion in *Chrebp* knockout mice than in WT mice, which is consistent with the effect of the genetic manipulation on hepatic MTTP expression (76). Thus, the lower MTP expression, together with the suppression of *de novo* lipogenesis, in the livers of *Chrebp* knockout mice may contribute to the lower hepatic VLDL secretion and plasma lipid concentrations (58, 76). Interestingly, hepatic *Chrebp* overexpression is also associated with lower plasma triglyceride concentrations, but a different mechanism is involved (32). Hepatic *Chrebp* overexpression is associated with higher hepatic triglyceride content but lower plasma triglyceride concentrations (32). The hepatic *Mtp* mRNA expression of mice with hepatic *Chrebp* overexpression is similar to that of control mice (32). Because hepatic *Chrebp* overexpression reduces the concentrations of VLDL-triglycerides and low-density lipoprotein-triglycerides (32), it has been suggested that lipoprotein lipase (LPL) might be activated by hepatic *Chrebp* overexpression. Consistent with this, we found that hepatic *Chrebp* overexpression increased hepatic *Fgf21* mRNA expression and the plasma FGF21 concentration (20, 32, 57). We also found that hepatic *Chrebp* overexpression was associated with lower hepatic mRNA and protein expression of ANGPTL3 (32). Because LPL is activated by FGF21 and suppressed by the ANGPTL3/ANGPTL8 complex, it is conceivable that high FGF21 and low ANGPTL3 might activate peripheral lipoprotein metabolism by increasing LPL activity, and thereby reducing plasma triglyceride concentration (32). Thus, ChREBP controls lipoprotein metabolism through the regulation of *de novo* lipogenesis, VLDL secretion, and the secretion of hepatokines that influence peripheral lipoprotein metabolism (**Figure 3**).

## Carbohydrate Response Element Binding Protein and Fatty Acid Esters of Hydroxyfatty Acids

**Figure 4:**
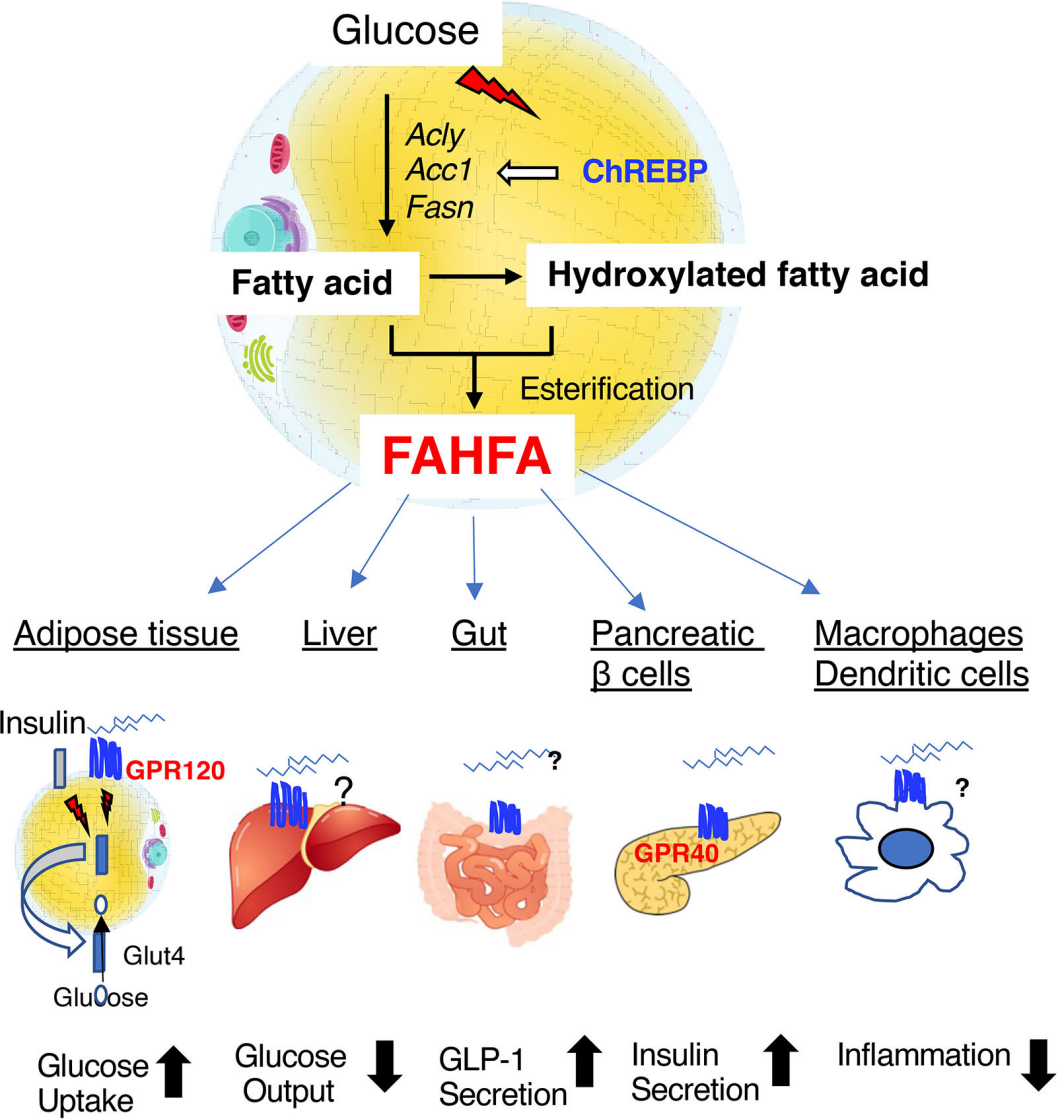
Fatty acid esters of hydroxyfatty acid (FAHFAs) are novel bioactive lipids that are produced in adipose tissue under the regulation of ChREBP. FAHFAs affect glucose uptake in adipose tissue, hepatic glucose output, GLP-1 secretion in the intestine, insulin secretion by pancreatic β-cells, and inflammation mediated by macrophages and dendritic cells by activating GPR120 (adipose tissue), GPR40 (pancreatic β-cells), and other unknown mediators. Abbreviations: GLP-1, glucagon-like peptide 1; *A*cly, ATP citrate lyase; *Acc1*, acetyl CoA carboxylase 1; *Fasn*, fatty acid synthase.

Fatty acid esters of hydroxyfatty acids (FAHFAs) are a recently discovered class of endogenous lipids that have anti-diabetic and anti-inflammatory properties (78–83). FAHFAs, and especially palmitic-acid-hydroxy-stearic-acid (PAHSA), have been found to be present in much higher concentrations in mice that overexpress GLUT4 in adipose tissue, which is a major site of FAHFA synthesis (78, 79). PAHSA is hydrolyzed by carboxyl ester lipase, mutations of which are known to cause maturity-onset diabetes of the young type 8 (80). In humans, FAHFAs can be found in serum, breast milk, meconium, and adipose tissue; and the serum PHASA concentration correlates with insulin sensitivity (81). It has been shown that PAHSA activates G-protein coupled receptor 120 (GPR120) to increase insulin-stimulated glucose uptake by adipocytes and glucagon-like-peptide-1 (GLP-1) secretion in the intestine (82). In addition, PAHSA activates G-protein coupled receptor 40 (GPR40) to increase glucose-induced insulin secretion (82) (**Figure 4**). PAHSA reduces adipose tissue inflammation and potentiates the insulin-induced suppression of hepatic glucose production (78, 79, 83). Whole-body *Chrebp* knockout mice have lower PAHSA concentrations in their adipose depots (78). Recently, adipose tissue-specific *Chrebp* knockout mice were also shown to have lower PAHSA concentrations in their serum and adipose tissue (83). Although the relationship between carboxyl ester lipase and ChREBP remain unclear, ChREBP regulates *de novo* lipogenesis in adipose tissue. Therefore, ChREBP may regulate PAHSA concentration *via* effects on biosynthetic and/or degradative pathways in adipose tissue. because a reduction in adipose *Chrebp* expression causes insulin resistance, the administration of PAHSA might be a means of ameliorating this defect, but further investigation is needed to clarify the mechanism involved.

## Carbohydrate Response Element Binding Protein Regulates B-Oxidation and Ketogenesis

β-oxidation and ketogenesis are upregulated in the fasting state to produce ketone bodies that are used as an energy source in peripheral tissues, including brain, heart, and muscle. Through β-oxidation, fatty acyl CoA is converted to acetyl CoA; and the resulting acetyl CoA is then converted to 3-hydroxybutyric acid (OHBA) or acetoacetyl CoA through ketogenesis. Although ChREBP is activated by feeding, it is also involved in β-oxidation and ketogenesis in the fasting state (30, 32, 76), which was suggested by the observation that hepatic *Chrebp* overexpression reduces plasma OHBA concentration and increases free fatty acid concentrations (32). Hepatic *Chrebp* overexpression causes an increase in the expression of *Acc2*, which catalyzes the conversion of acetyl CoA to malonyl CoA. Malonyl CoA reduces the entry of acyl CoA into mitochondria by inhibiting carnitine palmitoyl transferase-1 (84). Hepatic *Chrebp* overexpression also reduces the expression of acyl CoA oxidase (58). Therefore, it has been suggested that ChREBP activation suppresses β-oxidation and ketogenesis. However, *Chrebp* knockout mice have paradoxically low plasma OHBA concentrations, compared to their FFA concentrations, which suggests that ChREBP inhibition also suppresses β-oxidation and ketogenesis (30, 31, 58, 76). Recently, it was reported that peroxisome proliferator-activated receptor-α (PPARα) is required for the ChREBP-mediated transcriptional activation of the *Fgf21* gene (85). Although ChREBP and PPARα mRNA expression are reciprocally regulated in other tissues such brown adipose tissues (86), these results suggest that ChREBP may act in concert with PPARα to regulate gene transcription, even in the fasting state, to fine-tune β-oxidation and ketogenesis.

## Conclusions and Further Perspectives

The last two decades of research have revealed critical roles of ChREBP in lipid metabolism. ChREBP regulates hepatic lipogenesis *via* acetyl-CoA produced by the conventional pathway (glycolysis and fructolysis) and a novel pathway (conversion of gut microbiota-derived acetate). It also regulates lipoprotein metabolism in the liver and PAHSA production in adipose tissue. Furthermore, ChREBP regulates β-oxidation and ketogenesis in concert with PPARα. However, despite remarkable progress in understanding of the roles of ChREBP in these processes, a number of questions remain regarding its relationship with lipid metabolism. First, to what extent does gut microbiota-derived acetate induce hepatic triglyceride accumulation? Although ChREBP regulates *Acss2* mRNA, does acetate itself affect ChREBP transcriptional activity? Second, the role of ChREBP in cholesterol metabolism remains to be determined. *Chrebp* knockout mice have lower plasma concentrations of cholesterol, despite normal cholesterol synthesis in their livers, and the low plasma cholesterol concentrations may affect physiological processes, such as steroidogenesis in the adrenal gland. Third, the physiological and pathophysiological roles of ChREBP in the fasting state require more in-depth investigation. Because ChREBP is normally activated by glucose, it is conceivable that it regulates post-prandial lipid metabolism. However, it remains unclear why the ability of ChREBP to regulate β-oxidation and ketogenesis evolved. Finally, it remains unclear whether ChREBP suppression modifies the microbiota and lipid metabolism in humans. As discussed above, *Chrebp* knockout mice have an altered gut microbiota due to impaired fructose absorption from the gut (25). Consistent with this, it has been demonstrated that metformin treatment modifies the gut microbiota (87). AMP-activated protein kinase (AMPK) is known to be a major target of metformin, and it also regulates the activity of several transcription factors, such as SREBP-1c, PGC-1, FOXO1, and CREB. In addition, both AMPK and metformin reduce the transcriptional activity of ChREBP. Therefore, the effects of metformin on the microbiota might be mediated partly through ChREBP (88–90). Genome-wide scanning has also identified a human ChREBP homologue, *MLXIPL*, genetic variants of which are associated with plasma triglyceride concentration (91). Thus, it would be interesting to determine the effects of certain dietary habits (e.g., high fructose consumption) on the gut microbiota of individuals with *MLXIPL* variants (92). Further investigation is necessary to clarify the various physiological and pathophysiological roles of ChREBP in lipid metabolism.

## Author Contributions

All the authors (KI, KT, and DY) have made a substantial, direct intellectual contribution to the work, and approved it for publication.

## Funding

This work was supported by grants from Japan Society for the Promotion of Sciences (JSPS) [KAKENHI Grant 17K00850 and 20K11645 (to K. I.) and 17K09825 (to D.Y.)].

## Conflict of Interest

The authors declare that the research was conducted in the absence of any commercial or financial relationships that could be construed as a potential conflict of interest.
